# Comprehensive Analysis of Genomic and Expression Data Identified Potential Markers for Predicting Prognosis and Immune Response in CRC

**DOI:** 10.1155/2022/1831211

**Published:** 2022-07-30

**Authors:** Yongshan He, Xuan Dai, Yuanyuan Chen, Shiyong Huang

**Affiliations:** Department of Colorectal Surgery, Xinhua Hospital Affiliated to Shanghai Jiaotong University, School of Medicine, No. 1665 Kongjiang Road, Shanghai 200092, China

## Abstract

Colorectal cancer (CRC) is the most prevalent type of malignant tumor of the gastrointestinal tract. In the current study, we characterized the landscape of genomic alterations in CRC patients. Based on the results of whole-exome sequencing (WES), we identified 31 significantly mutated genes. Among them, several genes including TP53, KRAS, APC, PI3KCA, and BRAF were reported as significantly mutated genes in previous studies. In the current study, the most frequently mutated gene was TP53, which encodes tumor suppressor p53, affecting approximately 60% of CRC patients. In addition, we performed the expression profiles of significantly mutated genes between the normal group and tumor groups and identified 20 differentially expressed genes (DEGs); among them, CSMD3, DCHS2, LRP2, RYR2, and ZFHX4 were significantly negatively correlated with PFS. Moreover, consensus clustering analysis for CRC based on the expression of significantly somatic mutated genes was performed. In total, three subtypes of CRC were identified in CRC, including cluster1 (*n* = 453), cluster2 (*n* = 158), and cluster 3 (*n* = 9), based on expression level of significantly somatic mutated genes. Clinicopathological features analysis showed subtype C1 had the longest progression-free survival (PFS) with median time of 8.2 years, while subtypes C2 and C3 had 4.1 and 2.7 years of PFS, respectively. Moreover, we found three subtypes related to tumor infiltration depth, lymph node metastasis, and distant metastasis. Immune infiltration analysis showed the tumor infiltration levels of B cell native, T cell CD8+, T cell CD4+ memory activated, T cell gamma delta, NK cell resting, macrophage M0, macrophage M2, myeloid dendritic cell activated, mast cell activated, and mast cell resting significantly changed among the three groups, demonstrating the three subgroups classified by 22 somatically significantly mutated genes had a high capacity to differentiate patients with different immune statuses, which is helpful for the prediction of immunotherapy response of CRC patients. Our findings could provide novel potential predictive indicators for CRC prognosis and therapy targets for CRC immunotherapy.

## 1. Introduction

Colorectal cancer is the most prevalent type of malignant tumor of the gastrointestinal tract [1]. In 2020, colorectal cancer is the third most common cancer, with more than 193000 diagnosed cases [2], leading to the second cause of cancer-related deaths, with around 830000 fatalities. Studies have found that nearly 50% of colorectal cancer patients will eventually have distant metastases, and the resistance of colorectal cancer to chemotherapy drugs has led to treatment failure and tumor metastasis invasion [3]. Immunotherapy, in addition to surgery and chemotherapy, has been identified as a promising treatment for specific subtypes of CRC [4]. However, there were differences in prognosis amongst patients with the same disease stage, which were linked to distinct genetic abnormalities, underlining CRC's molecular heterogeneity.

The human immune system will go through three stages of tumor development: immune clearance, immune balance, and immune escape [5]. Based on this theory, immunotherapy plays a role to restore or enhance the antitumor effect of the immune system. With the development of tumor cytology, immunotherapy has been paid more and more attention in the treatment of malignant tumors and has achieved good efficacy in some subtypes of colorectal cancer patients [6]. Currently, the research related to immune checkpoint inhibitors is relatively mature and widely used in tumor immunotherapy. Immunotherapy drugs primarily target PD-1, PD-L1, and CTLA-4 [7]. At present, PD-1/PD-L1 inhibitors are relatively commonly used in the immunotherapy of colorectal cancer, and their efficacy is relatively favorable in patients with deficient mismatch repair (dMMR) or microsatellite instability-high (MSI-H) types, and the efficacy in patients with other subtypes needs to be further explored, in which the level of PD-L1 expression may have an impact on the efficacy [8]. However, mismatch repair proficient (MMR) or microsatellite stable (MSS) types account for the majority of patients with colorectal cancer. In recent years, some researchers have begun to focus on the immune escape mechanism of pMMR/MSS colorectal cancer, trying to convert its immune “cold environment” into a “hot environment.” Recent advances in genomics and bioinformatics have facilitated identification of new immune-related genes through cancer genome sequencing [9]. Therefore, it is promising to develop a novel genomic and expression-based classification of CRC with an improved clinical significance.

In this study, we compared gene expression differences between wild-type genes and mutant genes using expression profiles and genomic data, as well as performed Cox regression analysis. In addition, bioinformatics analysis was employed to investigate the clinical profile and distinct characteristics of immunogenicity of different subtypes of CRC. Our findings could provide novel potential predictive indicators and therapy targets for CRC immunotherapy.

## 2. Materials and Methods

### 2.1. Data Acquisition

TCGA database (https://portal.gdc.cancer.gov/) was used to extract mutational data, RNA-seq data, and clinical medical information of CRC patients. Our study included a total of 536 CRC samples, which included 536 CRC tumor samples and 536 adjacent normal samples.

### 2.2. Unsupervised Consensus Clustering Analysis

Using the Consensus Cluster Plus R package, we conducted an unsupervised consensus analysis [10]. In a brief, a graph of the consistency matrix based on the *k* value is displayed. Furthermore, for each *k*, the empirical cumulative distribution function plot shows a uniform distribution. The cluster consensus graph depicts the cluster consensus values for various *k* values. A higher cluster consensus value indicates a lower level of cluster stability. The average consensus value is drawn from a project and members of a consensus cluster are represented by a project consensus graph. Multiple project consensus values with varying *k* values are displayed in a project.

### 2.3. Immune Signature Analysis in Molecular Subtypes

The ESTIMATE algorithm was used to calculate expression scores for microenvironmental factors [11]. Tumor samples were analyzed with TIMER [12] for six tumor-infiltrating lymphocytes, including CD8+ T cells, dendritic cells, neutrophils, B cells, macrophages, and CD4+ T cells. Heatmaps were used to visualize the expression scores for immune signatures in different subgroups of CRC. Immune signatures and checkpoint gene expression levels were also examined across all molecular subtypes.

### 2.4. Differentially Expressed Gene Analysis

DEGs between different clusters of CRC were determined by Student's *t*-test, observing cutoff values |log2 (fold change)| > 1 and *p* values <0.05.

## 3. Results

### 3.1. The Landscape of Somatic Mutations of CRC

**Figure 1:**
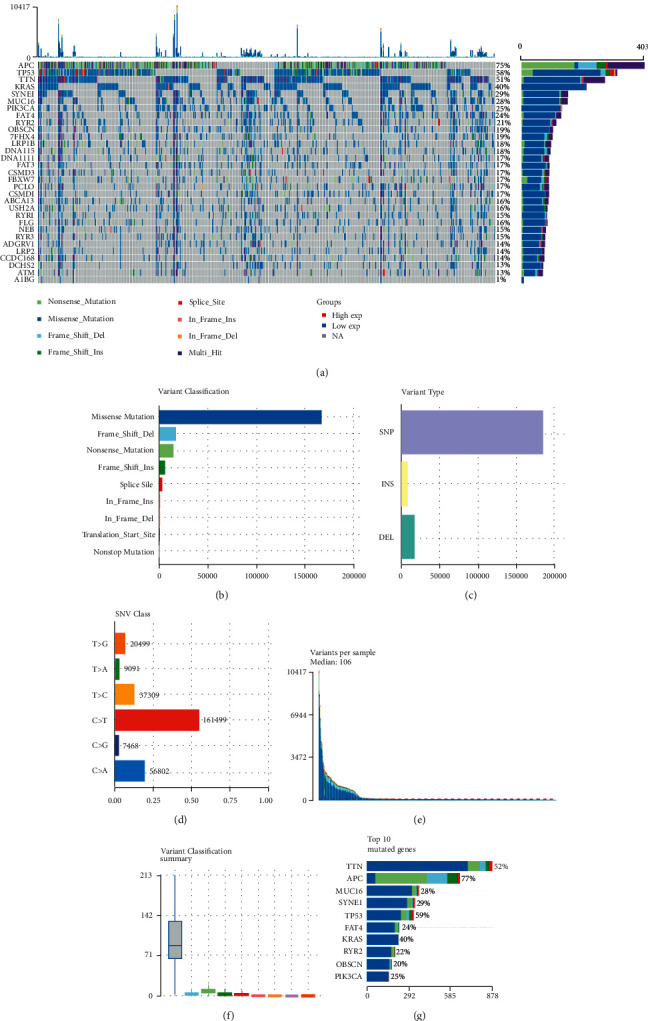
Landscape of somatic mutations of CRC. (a) Oncoplot and waterfall plot showing the somatic landscape of top 31 mutated genes in CRC. The distribution of variant classification (b), variant type (c), and SNV class (d) present. (e)-(f) Mutation load of each sample (variant classification type), and stacked bar graph shows the top ten mutated genes, including TTN, APC, MUC16, SYNE1, TP53, FAT4, KARS. RYR2, OBSCN, and PIK3CA.

536 WES samples data were conducted to analyze the landscape of somatic mutations of CRC. There were 525 samples (97.95%) altered in a total of 536 samples (Figure 1(a)). In addition, 31 genes were identified as significantly mutated genes including APC, TP53, KRAS, PIK3CA, TNN, SYNE1, MUC16, FAT4, RYR2, OBSCN, ZFHX4, LRP1B, DNAH5, DNAH11, FAT3, CSMD3, FBXW7, PCLO, CSMD1, ABCA13, USH2A, RYR1, FLG, NEB, RYR3, ADGRV1, LRP2, CCDC168, DCHS2, ATM, and A1BG (Figure 1(a)). The variant classification can be divided into 9 types, among which missense mutations account for the majority (Figures 1(b) and 1(c)). The predominant somatic mutation types were *C* > *T* (Figure 1(d)). The median variants per sample were 106. TTN, APC, MUC16, SYNE1, TP53, FAT4, KARS, RYR2, OBSCN, and PIK3CA were the top 10 mutant genes (Figures 1(e) and 1(g)).

### 3.2. Identification of Survival-Associated Somatic Mutated Genes

**Figure 2:**
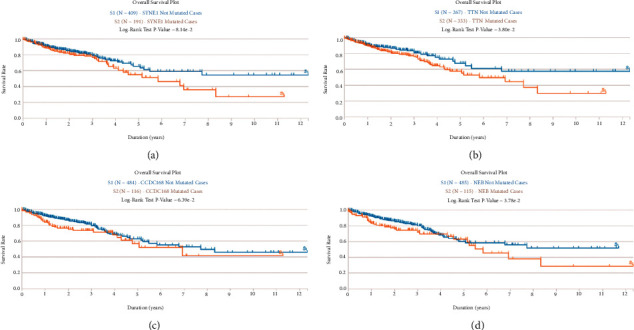
Identification of survival-associated somatic mutated genes. The CRC patients with SYNE1 (a), TNN (b), CCDC168 (c), and NEN (d) mutations had a shorter overall survival time.

**Figure 3:**
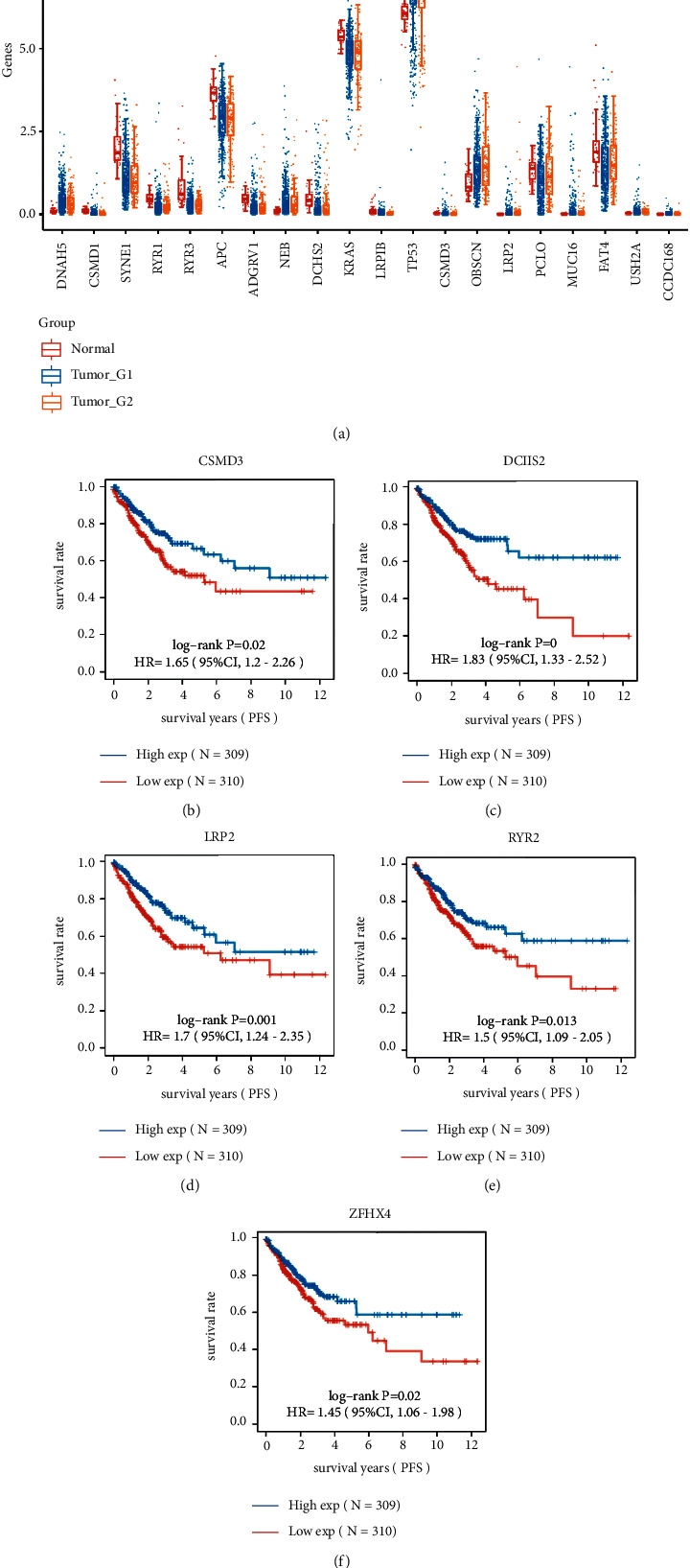
The dysregulation of somatically mutated genes was correlated to the prognosis of CRC. (a) 20 genes dramatically changed between the normal colon tissue and CRC group. Among them, DNAH5, TP53, OBSCN, LRP2, NEB, PCLO, MUC16, USH2A, and CCDC168 significantly upregulated. CSMD1, SYNE1, RYR1, RYR3, APC, ADGRV1M, DCHS2, KRAS, LRP1B, and FAT4 significantly downregulated. High expression of CSMD3 (b), DCHS2 (c), LRP2 (d), RYR2 (e), and ZFHX4 (f) significantly negatively correlated with shorter PFS in CRC.

To investigate the link between somatic mutations and CRC prognosis, 31 significantly mutated genes were divided into two groups, respectively. We identified 4 genes SYNE1, TNN, CCDC168, and NEN mutations were significantly related to short overall survival (Figures 2(a)–2(d)). In addition, we performed the expression profiles of significantly mutated genes in one normal group and two tumor groups. We found that 20 genes were dramatically changed between the normal colon tissue and tumor group (Figure 3(a)). Among them, DNAH5, TP53, OBSCN, LRP2, NEB, PCLO, MUC16, USH2A, and CCDC168 were significantly upregulated. CSMD1, SYNE1, RYR1, RYR3, APC, ADGRV1M, DCHS2, KRAS, LRP1B, and FAT4 were significantly downregulated (Figure 3(a)) Furthermore, the relationship between the expression of somatically mutated genes and progression-free survival (PFS) in CRC was investigated. We identified high expressions of CSMD3, DCHS2, LRP2, RYR2, and ZFHX4 were significantly negatively correlated with PFS in CRC (Figures 3(b)–3(f)).

### 3.3. Consensus Clustering Analysis for CRC Based on the Expression of Significantly Somatic Mutated Genes

**Figure 4:**
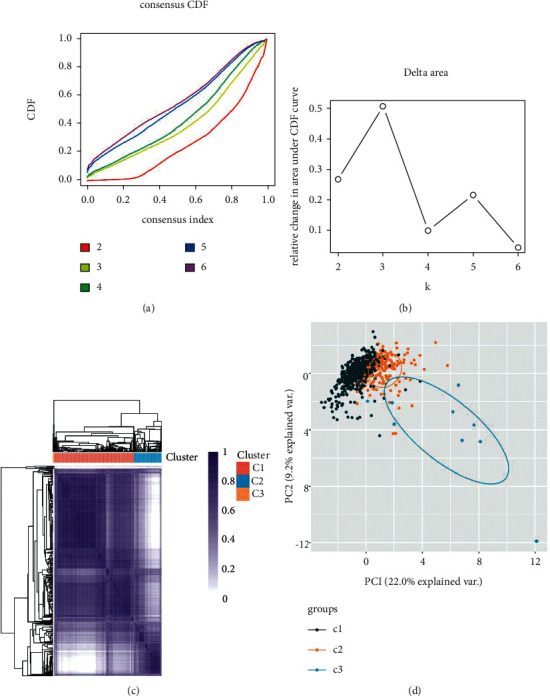
Consensus clustering analysis for CRC based on the expression of significantly somatic mutated genes. (a) The cumulative distribution function analysis of CRC subtypes. (b) Relative change in the area under the CDF curve (CDF delta area) presented. At *k* = 3, the analysis had the best delta area scores. (c) Three subtypes of CRC identified in CRC samples. (d) The PCA analysis of three subtypes of CRC performed.

**Figure 5:**
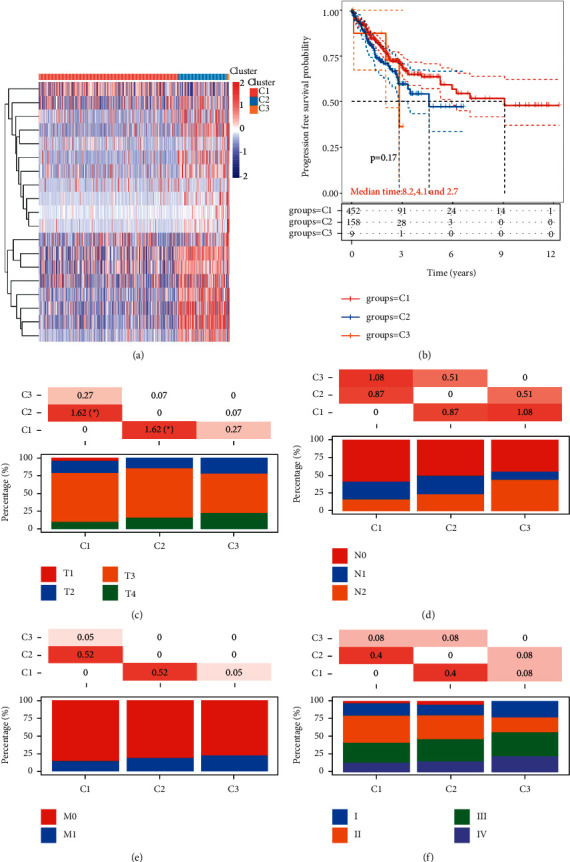
Clinicopathological features analysis of somatically mutated genes related subgroups of CRC. (a) Heatmap showing the expression levels of somatically mutated genes in subgroups of CRC. (b) Kaplan–Meier survival analysis of the correlation between subgroups and PFS time in CRC. (c) The distribution analysis of T stage in different subgroups of CRC. (d) The distribution analysis of N stage in different subgroups of CRC. (e) The distribution analysis of M stage in different subgroups of CRC. (f) The distribution analysis of grades in different subgroups of CRC.

Consensus clustering was performed using the Consensus Cluster Plus R program. At consensus index, with *k* = 3, the cumulative distribution function has the lowest rangeability (Figure 4(a)). At *k* = 3, the analysis had the best delta area scores (Figure 4(b)). In total, three subtypes of CRC were identified in 620 CRC samples, including cluster1 (*n* = 453), cluster2 (*n* = 158), and cluster 3 (*n* = 9) based on expression level of significantly somatic mutated genes (Figures 4(c) and 4(d)). Our data show that clustered subtypes defined by the expression level of somatically mutated genes are closely related to the heterogeneity of CRC patients (Figure 5(a)).

### 3.4. Clinicopathological Features Analysis of Somatically Mutated Genes Related Subgroups of CRC

Unsupervised clustering based on subgroups formed from mutational signatures and critical gene changes was carried out to establish genomic categorization of CRC linked with patients' clinical and pathological features and progression-free survival. As shown in Figure 5(b), subtype C1 had the longest PFS with a median time of 8.2 years, while subtypes C2 and C3 had 4.1 and 2.7 years PFS, respectively. In addition, we found that three subtypes related to the grade and metastasis stage. Subtype C1 contains more early stage, subtype C2 is intermediate malignancy, while subtype 3 has the greatest malignancy (Figures 5(c)–5(f)).

### 3.5. Association between Immune Infiltration and Genomic Consensus Cluster in CRC

**Figure 6:**
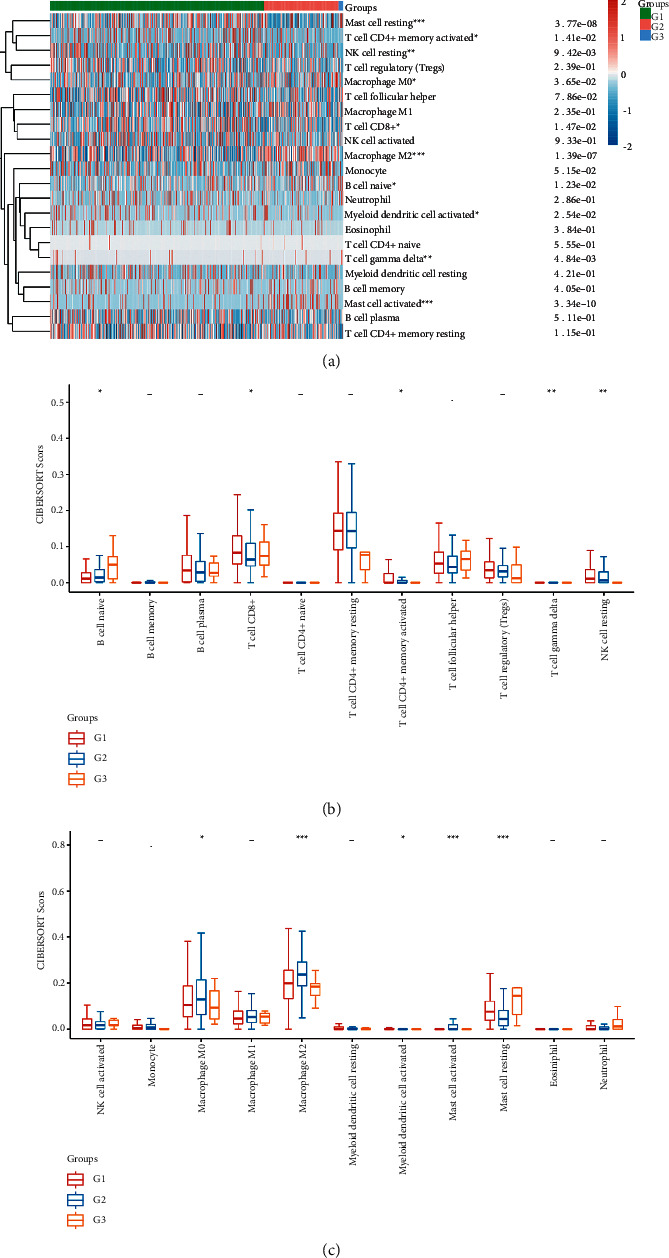
Association between immune infiltration and genomic consensus cluster in CRC. (a) Heatmap analysis showing immune infiltration of immune cells in three subgroups by the CIBERSORT algorithm. (b) Box-plot analysis showing immune infiltration levels in three subgroups by the CIBERSORT algorithm.

In subtyping analysis, we compared the immune infiltration of the three subgroups by the CIBERSORT algorithm. We found that B cell native, T cell CD8+, T cell CD4+ memory activated, T cell gamma delta, NK cell resting, macrophage M0/M2, myeloid dendritic cell activated, mast cell activated, and mast cell resting had differences among the three groups (Figure 6).

Additionally, a higher number of B cell native were produced in subtype C3 than in other subtypes; T cell CD4+ memory resting/activated and NK cell resting were significantly lower in C3 than in C1 and C2 subtypes; macrophage M0/M2, myeloid dendritic cell activated, and mast cell activated were significantly higher in C2 than in C1 and C3 subtypes; interestingly, mast cell resting was significantly lower in C2 than in C1 and C3 subtypes (Figures 6(a) and 6(b)).

## 4. Discussion

In the current study, we characterized the landscape of genomic alterations in CRC patients. Based on the results of WES, we identified 31 significantly mutated genes. Among them, several genes including TP53, KRAS, APC, PI3KCA, and BRAF were reported as significantly mutated genes in previous studies. In the current study, the most frequently mutated gene was TP53, which encodes tumor suppressor p53, affecting approximately 60% of CRC patients. A previous study showed that loss of p53 transcriptional activity leads to uncontrolled cell growth in various organs, including the colon [13]. In addition, KRAS mutations are the main intestinal cancer markers. Studies showed that 30% of human malignant tumors were related to KRAS gene mutation. The mutated KRAS is activated and unable to create normal RAS protein, causing the intracellular signal to be disrupted, resulting in uncontrolled cell proliferation and cancer [14]. Furthermore, mutations in the APC gene are the molecular pathological basis of adenomatous polyposis (FAP) and play a crucial role in the development of sporadic colorectal cancer [15]. APC protein deficiency results in *β*-catenin accumulation in the cytoplasm; this leads to sustained transcriptional activation of TCF-mediated genes and promotes colorectal cancer progression [16]. BRAF mutations are observed in 8–12% of patients with advanced disease, and a valine amino acid substitution (V600E) in exon 15 is the most common alteration. BRAF mutations in CRC cause MAPK/ERK signaling abnormal activation, which affects cell growth and differentiation pathways [17]. Mutations in the PIK3CA gene cause the creation of an altered p110 subunit, which permits PI3K to hypersignal without being regulated, triggering malignant cell actions such as proliferation and migration [18]. In addition to these commonly mutated genes, genomic mutations in SYNE1, TTN, NEB, and CCDC168 were found to be associated with a poor prognosis of colorectal cancer in this study. SYNE1 encodes a spectrin repeat-containing protein Nesprin-1 that localizes to the nuclear membrane. Mutation of SYNE1 in exonic rs9479297 leads to the upregulation of proteins SYNE1, which may contribute to cell proliferation and migration in hepatocellular and transitional cell carcinoma [19]. Loss of Nesprin-1 in CRC cells may alter cell destiny, contributing to carcinogenesis. In our study, somatic-mutated SYNE1 cases displayed a worse overall survival. Djulbegovic et al. demonstrated that in ocular surface squamous neoplasia, this gene mutation is the most common genetic abnormality. TTN mutations are also linked to resistance to topical interferon alpha-2b (IFN-2b) therapy, which promotes chromosomal instability, oncogenesis, and a changed response to IFN-2b treatment. Xie et al. discovered that TTN mutation was enriched in samples with high immunostimulatory signatures and that the mutation load within TTN implies high TMB status, which is consistent with our finding that somatically mutated TTN cases had a lower overall survival [20]. NEB encodes nebulin, a giant protein component of the cytoskeletal matrix. In taxol-resistant ovarian cancer cells, NEB along with DCDC2, ANKRD18B, ALDH1A1, and ITGBL1 were overexpressed suggesting the involvement of these AR-related genes in taxol resistance. NEB was also identified as a potential cancer-related gene, but research into NEB in CRC is still in its early stages. In our study, we demonstrate that NEB-mutated cases are associated with the worse overall survival in CRC patients.

In addition, we performed the expression profiles of significantly mutated genes in one normal group and two tumor groups; we identified 20 DEGs; among them CSMD3, DCHS2, LRP2, RYR2, and ZFHX4 were significantly negatively correlated with PFS. Lu et al. discovered that CSMD3 is linked to tumor mutation burden and immune infiltration in ovarian cancer patients [21] and was identified as a potential driver gene in prostate adenocarcinoma. However, it is unclear whether CSMD3 is a driver protein in CRC and how CSMD3 contributed to tumorigenesis. DCHS2 is a big protein with numerous cadherin domains that are thought to play a role in cell adhesion. This gene may be relevant in the stomach and colorectal malignancies with high microsatellite instability, according to genome-wide association studies. It suggests that DCHS2 may relate to the immune response of MSI-H [22]. LRP2/megalin was one of the first endocytic cargos identified for the Dab2 adaptor and has been found related to poor prognosis in various cancer. In thyroid tumors, LRP2 mediated the suppressive effect of metformin on cancer proliferation by blocking JNK signaling [23]. LRP2/megalin expression influences melanoma cell growth and survival rates in frequently acquired melanoma tumors [24]. In addition, LRP2 was reported to relate to fibrosis-associated diseases and cancer through the TGF-*β* pathway. Furthermore, RYR2 is an important player in steroid metabolism and cancer research. Several studies uncovered that RYR2 mutation played a positive side in tumor prognosis. Wei et al. found that RYR2 3′UTR polymorphisms remained significant in the genetic susceptibility of progesterone receptor positive breast cancer [25]. Liu et al. found the RYR2 mutation was linked to a greater TMB and a better clinical outcome by enhancing the antitumor immune response in breast cancer and esophageal adenocarcinoma [26]. In nonsmall cell lung cancer, RYR2 mutation may prolong survival via downregulation of DKK1 and upregulation [27]. In this study, we found that RYR2 expression was negatively related to the PFS in CRC. We speculate that the heterogeneity that exists between tumors causes RYR2 to exhibit different functions in colorectal cancer or due to the insufficient sample size of the analyzed data. ZFHX4 is a 397 kD putative transcription factor with 4 homeodomains and 22 zinc fingers that were discovered lately [28]. Chudnovsky et al. reported ZFHX4 interacts with CHD4 to govern the glioblastoma tumor initiating cell state [28]. ZFHX4 is also found as a prognostic factor for ovarian serous cystadenocarcinoma, esophageal cancer, and lung adenocarcinoma. Understanding the role of pivotal genetic variations in CRC cancer development and growth is a promising direction for future research.

CRC is a highly heterogeneous disease, and clinically similar tumors with similar pathology differ significantly in terms of treatment response and patient survival. Pathologic staging is critical to the efficacy of biological therapy for CRC, and the traditional classification of CRC does not fully reflect tumor heterogeneity and cannot accommodate the needs of modern cancer therapy. The concept of molecular classification is to shift the classification of tumors from morphological to molecular characteristics through comprehensive molecular analysis techniques. Afterward, with the in-depth study of the mechanism at the molecular level and the advancement of sequencing technology, the molecular typing methods of tumors have been expanded from relying on single or few markers detection to the stage of spectroscopic typing, which has led more researchers to perform molecular typing of CRC. However, there is no uniform standard for molecular typing of CRC for now. After analyzing 18 different CRC gene expression datasets, the International CRC subtyping collaborative (CRCSC) established 4 CRC molecular signature consensus subgroups (CMS) in 2015, including CMS1, CMS2, CMS3, and CMS4 [29]. Among them, CMS1 (MSI immune subtype) is distinguished by MSI, BRAF mutation, high CpG island methylator phenotype (CIMP), immune infiltration, and poor survival; CMS2 (typical) has a high somatic copy number alterations (SCNA) level; CMS3 (metabolic) is characterized by low SCNA and CIMP, KRAS mutation, and metabolic dysregulation; CMS4 (mesenchymal) is characterized by high SCNA, stromal infiltration, TGF activation, angiogenesis, and short survival. CMS typing is currently considered to be the most convincing method for colorectal cancer staging, and many researchers have conducted studies on CRC-targeted therapy based on this method. In this study, we identified the CRC subtype associated with the PFS. As shown in Figure 5(b), subtype C1 had the longest PFS with a median time of 8.2 years, while subtypes C2 and C3 had 4.1 and 2.7 years PFS, respectively. Patients' clinicopathological staging was also related to the CRC subtype that we identified.

Following the determination of the three subtypes based on the expression of CRC-related mutated genes, the immune infiltration of the three groups was evaluated by using the CIBERSORT method. We demonstrated that B cell native, T cell CD8+, T cell CD4+ memory activated, T cell gamma delta, NK cell resting, macrophage M0, macrophage M2, myeloid dendritic cell activated, mast cell activated, and mast cell resting had differences among the three groups. According to the findings, the three subgroups classified by 22 somatically significantly mutated genes had a high capacity to differentiate patients with different immune statuses, which is helpful for the prediction of immunotherapy response of CRC patients.

This study still has some limitations. First, despite the data from CRC patients used in this study being extracted from TCGA, the sample size was still small; second, despite the obvious clinical significance of the newly proposed subtypes, little is known about their underlying mechanism. As a result, we must investigate the molecular mechanisms of the three subtypes. Third, there is no validation of the functions of hub genes.

Finally, the current investigation confirmed the predictive significance of somatically altered genes and offered a novel genomic categorization with clinical relevance. This discovery presented a foundation for CRC research and molecular classification of CRC types to guide precision therapy by interpreting genomic data.

## Data Availability

The data used to support the findings of this study are available from the corresponding author upon request.

## Abbreviations

CRC: Colorectal cancer
WES: Whole exosome sequencing
DEGs: Differentially expressed genes
PFS: Progression-free survival
DMMR: Deficient mismatch repair
MSI-H: Microsatellite instability-high
MSS: Microsatellite stable
pMMR: Mismatch repair proficient
FAP: Familial adenomatous polyposis
CMS: CRC molecular signature consensus subgroups
TMB: Tumor burden
CIMP: CpG island methylator phenotype
SCNA: Somatic copy number alterations.

## References

[1] Chen H., Zheng X., Zong X. (2021). Metabolic syndrome, metabolic comorbid conditions and risk of early-onset colorectal cancer. *Gut*.

[2] Xi Y., Xu P. (2021). Global colorectal cancer burden in 2020 and projections to 2040. *Translational Oncology*.

[3] Filip S., Vymetalkova V., Petera J. (2020). Distant metastasis in colorectal cancer patients-do we have new predicting clinicopathological and molecular biomarkers? a comprehensive review. *International Journal of Molecular Sciences*.

[4] Golshani G., Zhang Y. (2020). Advances in immunotherapy for colorectal cancer: a review. *Therapeutic Advances in Gastroenterology*.

[5] Gonzalez H., Hagerling C., Werb Z. (2018). Roles of the immune system in cancer: from tumor initiation to metastatic progression. *Genes and Development*.

[6] Ventola C. L. (2017). Cancer immunotherapy, part 2: efficacy, safety, and other clinical considerations. *Pharmacy and Therapeutics*.

[7] Relecom A., Merhi M., Inchakalody V. (2021). Emerging dynamics pathways of response and resistance to PD-1 and CTLA-4 blockade: tackling uncertainty by confronting complexity. *Journal of Experimental & Clinical Cancer Research*.

[8] Oliveira A. F., Bretes L., Furtado I. (2019). Review of PD-1/PD-L1 inhibitors in metastatic dMMR/MSI-H colorectal cancer. *Frontiers in Oncology*.

[9] Chen H., Wu J., Lu L. (2020). Identification of hub genes associated with immune infiltration and predict prognosis in hepatocellular carcinoma via bioinformatics approaches. *Frontiers in Genetics*.

[10] Wilkerson M. D., Hayes D. N. (2010). Consensusclusterplus: a class discovery tool with confidence assessments and item tracking. *Bioinformatics*.

[11] Liu Q., Fang Y., Wang J. (2020). Estimate algorithm is not appropriate for inferring tumor purity and stromal and immune cell admixture in hematopoietic or stromal tumors. *Cancer Immunology Immunotherapy*.

[12] Li T., Fan J., Wang B. (2017). TIMER: a web server for comprehensive analysis of tumor-infiltrating immune cells. *Cancer Research*.

[13] Chen J. (2016). The cell-cycle arrest and apoptotic functions of p53 in tumor initiation and progression. *Cold Spring Harb Perspect Med*.

[14] Jancik S., Drabek J., Radzioch D., Hajduch M. (2010). Clinical relevance of KRAS in human cancers. *Journal of Biomedicine and Biotechnology*.

[15] Narayan S., Roy D. (2003). Role of APC and DNA mismatch repair genes in the development of colorectal cancers. *Molecular Cancer*.

[16] Morin P. J., Sparks A. B., Korinek V. (1997). Activation of beta-catenin-Tcf signaling in colon cancer by mutations in beta-catenin or APC. *Science*.

[17] Smiech M., Leszczynski P., Kono H., Wardell C., Taniguchi H. (2020). Emerging BRAF mutations in cancer progression and their possible effects on transcriptional networks. *Genes*.

[18] Ligresti G., Militello L., Steelman L. S. (2009). PIK3CA mutations in human solid tumors: role in sensitivity to various therapeutic approaches. *Cell Cycle*.

[19] Chu Y. D., Kee K. M., Lin W. R. (2021). SYNE1 exonic variant rs9479297 contributes to concurrent hepatocellular and transitional cell carcinoma double primary cancer. *Biomedicines*.

[20] Xie X., Tang Y., Sheng J. (2021). Titin mutation is associated with tumor mutation burden and promotes antitumor immunity in lung squamous cell carcinoma. *Frontiers in Cell and Developmental Biology*.

[21] Lu N., Liu J., Xu M. (2021). CSMD3 is associated with tumor mutation burden and immune infiltration in ovarian cancer patients. *International Journal of General Medicine*.

[22] An C. H., Je E. M., Yoo N. J., Lee S. H. (2015). Frameshift mutations of cadherin genes DCHS2, CDH10 and CDH24 genes in gastric and colorectal cancers with high microsatellite instability. *Pathology and Oncology Research*.

[23] He Y., Cao L., Wang L., Liu L., Huang Y., Gong X. (2020). Metformin inhibits proliferation of human thyroid cancer TPC-1 cells by decreasing LRP2 to suppress the JNK pathway. *OncoTargets and Therapy*.

[24] Andersen R. K., Hammer K., Hager H. (2015). Melanoma tumors frequently acquire LRP2 /megalin expression, which modulates melanoma cell proliferation and survival rates. *Pigment Cell Melanoma Research*.

[25] Wei Y., Wang X., Zhang Z. (2020). Impact of NR5A2 and RYR2 3′UTR polymorphisms on the risk of breast cancer in a Chinese han population. *Breast Cancer Research and Treatment*.

[26] Liu Z., Liu L., Jiao D. (2021). Association of RYR2 mutation with tumor mutation burden, prognosis, and antitumor immunity in patients with esophageal adenocarcinoma. *Frontiers in Genetics*.

[27] Ren W., Li Y., Chen X. (2022). RYR2 mutation in non-small cell lung cancer prolongs survival via down-regulation of DKK1 and up-regulation of GS1-115G20.1: a weighted gene Co-expression network analysis and risk prognostic models. *IET Systems Biology*.

[28] Chudnovsky Y., Kim D., Zheng S. (2014). ZFHX4 interacts with the NuRD core member CHD4 and regulates the glioblastoma tumor-initiating cell state. *Cell Reports*.

[29] Guinney J., Dienstmann R., Wang X. (2015). The consensus molecular subtypes of colorectal cancer. *Nature Medicine*.

